# Vaccination Testimony in April Number

**Published:** 1898-06

**Authors:** M. R. Leverson


### VACCINATION TESTIMONY IN APRIL NUMBER.

M. R. LEVERSON, M. D.

That portion of this abstract of the testimony taken by the Royal British Commission, which appeared in the April number of THE HOMŒOPATHIC PHYSICIAN, furnishes a striking illustration of the lack of the logical faculty which distinguishes the medical profession as represented by its most prominent members in England.

Not only Dr. Cory, who was testifying, but each of the medical members of the Commission, with the single exception of Dr. Collins, took for granted that the differences which were found to occur between the phenomena of primary vaccination and those of re-vaccination were due to residual effects of the former.

And yet there is one difference in the circumstances attending the operation of so fundamental a character that it is difficult to understand how so vicious an error could be fallen into by men of ordinary common sense, not to speak of claimants to scientific reputation.

Re-vaccination in nearly all cases is performed upon persons of much more mature age than primary vaccination; and, other things equal, the differences noted in the progress and results of primary and re-vaccination are just as likely to be due to the difference of age incidence as to any other factor of the problem.

But there is strong evidence which points to age incidence as the more probable cause. It was a fact well known to Dr. Cory and to the medical members of the Commission that primary vaccination proves unsuccessful in a much larger proportion of adults than of children, and this striking fact produced no effect upon the reasoning powers of Dr. Cory and the majority of the Commission!

But if it be true that the difference in progress and results between re-vaccination and primary vaccination is due in any degree whatever to "residual effects of primary vaccination," then this fact affords further and positive evidence of the baneful effects produced by the primary vaccination.

If the statement be true, it must mean that the blood of the victim of every successful vaccination is in an *abnormal* condition—*i. e.*, in an unhealthy one. And that this is the effect of vaccination and its deplorable effects upon the race there will be abundant reason for suspecting as we proceed with the testimony.

That "the parallelism as regards effects does not go very far" between cow-pox and syphilis, as said by Dr. Creighton in reply to Lord Herschel's question (5,575), is generally true of inoculated cow-pox where the "morbid poison" has been subjected to "management," but its normal virulence sometimes breaks out, and in such cases the parallelism as regards effects goes as far almost as identity.

The secondary symptoms of cow-pox as given in the following table are taken from descriptions of its effects given by advocates of vaccination in their works, perhaps inadvertently—when they were not concerned in "preserving vaccination from reproach."

I have a right to claim that this pathological table has for its support "the concensus of medical opinion," at least far more than vaccination ever had. For months before the meeting of the American Association of Physicians and Surgeons, held at Indianapolis in January, 1896, that table was circulated among the best known advocates of vaccination in Europe and North America, as well as among the Boards of Health of nearly every State and large city of America, accompanied by a letter from the Secretary of the Association, informing them that that table would be presented and sustained by the editor of the abstract of the Royal British Commission testimony now being published in THE HOMŒ-

### PATHOLOGICAL DIAGNOSTIC TABLE.

[From the Monograph on the pathology of Vaccination, read by Dr. M. R. Leverson, before the American Association of Physicians and Surgeons, at their annual meeting commencing January 15th, 1896.]

| SMALL-POX. | COW-POX. | GREAT-POX OR SYPHILIS. |
|---|---|---|
| | PRIMARY LESION. | |
| 1. Eruption general, superficial. | 1. Eruption local, deep, in the corium of skin or subcutaneous tissue, or in the mucous membrane. | 1. Eruption local, deep, in the corium of skin or subcutaneous tissue, or in the mucous membrane. |
| 2. Constitutional or general symptoms precede the eruption and are relieved on its appearance. | 2. Constitutional symptoms do not precede but follow the eruption in all cases. | 2. Constitutional symptoms do not precede but follow the eruption in all cases. |
| 3. Pustules of various kinds, irregular, elevated, generally perforated by a hair, induration, if any, very slight, no tendency to a gnawing ulceration of the skin. | 3. Pustule* always the same, round, centrally depressed, margin indurated and not perforated by a hair, has a cellular membrane at floor, tendency to a gnawing ulceration. | 3. Pustule* always the same, scooped out, deep funnel-shaped with sloping edges often elevated, not perforated by a hair, has a fungoid membrane at floor, tendency to a gnawing ulceration. |
| 4. The fluid is contained in two chambers a superficial and a deep, which communicate around the edges of the separating membrane; it is carried with the dust of the air. | 4. The fluid is contained in a single chamber, reticulated, is non-volatile and is communicated only by immediate contact with an abraided surface. | 4. Absolutely the same as the cowpox. |
| 5. The poison of small pox, like that of cholera, is produced spontaneously in certain seasons in unhealthy and filthy localities; where it is propagated by contagion at a distance or by contact. | 5. The poison is never produced spontaneously. It is always the result of direct contact between a vaccinated or syphilized subject and an abraided skin or a mucous membrane. | 5. The poison is not produced spontaneously. It must either come in contact with an abraided skir or with the mucous membrane. |
| 6. The small-pox pustules *leave no scar* if properly treated. It is bad treatment and exposure to light during their evolution which is the cause of the scarification, formerly so common. | 6. The cowpox leaves a special foveated scar, owing to its having attacked the whole skin, and spreads out in different directions by erosion of the skin. | 6. Similar to the scar of cowpox, but is generally slight and varies more in character. |

**i. e.*, The chancre.

| | | |
|---|---|---|
| 7. The small-pox eruption does not affect the lymphatic system. | 7. The cowpox poison permeates the lymphatic channels and ganglia, causing cutaneous inflammation, buboes and abscesses in various parts of the body except when greatly attenuated as by passing through the tissues of different subjects. | 7. Absolutely the same as the cowpox. |
| 8. Infectious. | 8. Not infectious. | 8. Not infectious. |
| 9. Inoculable. | 9. Inoculable. | 9. Inoculable. |
| 10. Both Sexes. | 10. Cow only.* | 10. Both sexes. |
| 11. The small-pox is epidemic; taking its rise in filthy localities, and can be arrested in its march by disinfectants and *cleanliness*. | 11. Cowpox is independent of time and place; it is sporadic, accidental, individual, communicated only by direct inoculation. | 11. Absolutely the same as cowpox. |
| | SECONDARY SYMPTOMS. | |
| 12. None. The pits are the scars of the eruption, consequent on bad treatment; sometimes blindness follows: but this is a consequence of the acute attack and is not a "secondary" symptom; it occurs through failure to observe and give special treatment for a pustule appearing on the cornea; sometimes pulmonary troubles follow, but these also result from bad treatment. | 12. Sometimes buboes which discharge an ichorous pus, it poisons the blood and causes enlarged glands. | 12. Buboes, sometimes discharging an ichorous pus, it poisons the blood and causes enlarged glands. |
| | 13. Snuffles. | 13. Snuffles. |
| | 14. Thrush. | 14. Thrush. |

* Cowpox may be inoculated on the bull. But so far as I have been able to learn no case of "spontaneous" cowpox has ever been observed in the bull.

SECONDARY SYMPTOMS—*Continued.*

| | |
|---|---|
| 15. Phagedenic sores. | 15. Phagedenic sores. |
| 16. Eruptions and abscesses on genitals in infants. | 16. Eruptions and abscesses on genitals in infants. |
| 17. Nodes in the head. | 17. Nodes in the head. |
| 18. Opthalmia. | 18. Opthalmia. |
| 19. Alopecia. | 19. Alopecia. |
| 20. Cephalalgia. | 20. Cephalalgia. |
| 21. Dentition delayed in children, with production of the so-called syphilitic teeth. | 21. Dentition delayed in children, with production of the so-called syphilitic teeth. |
| 22. Dermatitis gangrenosa infantum. | 22. Dermatitis of every kind; rarely gangrenosa. |
| 23. Erythema, especially papulatum. | 23. Erythema. |
| 24. Impetigo. | 24. Impetigo. |
| 25. Eczema of all kinds. | 25. Eczema. |
| 26. Herpes. | 26. Herpes. |
| 27. Ready fracture and difficult healing of bone, also probably* caries in some cases. | 27. Caries of bone; many authorities regard this as a tertiary symptom only, a result of bad treatment and not properly a secondary of syphilis. |
| 28. Photophobia. | 28. Photophobia. |
| 29. Insanity, probably.* | 29. Insanity. |
| 30. Scrofula. | 30. Scrofula. |
| 31. Macula roseola. | 31. Macula roseola. |
| 32. Condylomata. | 32. Condylomata. |

| | | |
|---|---|---|
| | 33. Mucous patches on tonsils, tongue and lips tending to ulceration. | 33. Mucous patches on **tonsils**, tongue and lips tending to **ulceration**. |
| | 34. Acne. | 34. Acne. |
| | 35. Bronchitis. | 35. Bronchitis. |
| | 36. Tuberculosis, probably.* | 36. Tuberculosis. |
| | 37. Arrest of development. | 37. Arrest of development. |
| After recovery from the attack, the patient is restored to normal health. | 38. Not only is the cow-pox no preventive against small-pox, but those subjected to it are predisposed to attacks of epidemics or infectious disorders, such as typhoid, cholera, small-pox. This is conclusively established by statistics: Of Germany by Dr. Oidtmann, of Switzerland by Prof. Dr. Vogt, of England by Alfred R. Wallace, Wm. Tebb, and Alex. Wheeler, and among the workmen and prisoners of Belgium by Dr. Hubert Boens. | 38. Absolutely the **same as in** cowpox. |
| | 39. The name of vaccinides has been given to the different skin diseases resulting from vaccination. | 39. The name syphilides has been given to the different skin diseases following upon syphilis, and |
| | 40. Vaccinides and syphilides are cured by the same treatment. | |
| Small-pox will not beget cowpox or syphilis. | 41. Cowpox will not beget small-pox. | 41. Syphilis will not beget small-pox. |

* The word "probably" is used because the evidence is that only of the great increase of insanity and tuberculosis following the increase of vaccination, such increase being proportionately greater among the vaccinated than among the unvaccinated.

Continue, gentlemen, to vaccinate if you choose, and *because you make money by it;* but never forget, pseudo scientists and false physicians that you are, that while you sow vaccine among the people, THEY REAP THE POX! [Adapted from the allocution of Dr. Hubert Boens, of Brussels.]

OPATHIC PHYSICIAN, and they were invited to be prepared to contest the same. No one appeared to do so.

The table was also published in the transactions of the Medico-Legal Society of New York, and has been widely circulated in a pamphlet in which the proceedings of the Medico-Legal Society of New York, of November, 1896, were fully published.

Several sessions of the American Association of Physicians and Surgeons have since been held, preceded in some cases with urgent invitations to medical men, and especially to Boards of Health and well known advocates of vaccination, to dispute the accuracy of the table. It remains undisputed. Therefore, the author claims it must now be regarded as representing "the concensus of medical opinion" upon the subject as much as any doctrine which has ever been upheld by that very intangible entity or imaginary force.

[P. S.—In a recent number of the *Medical Argus*, published since the foregoing note was written, the editor of that journal makes the same claim for my table, viz., that it must now be taken as representing "the concensus of medical opinion" upon the subject. M. R. L.]

---

RAMPANT ORGANOTHERAPY.—A writer in the *Chicago Medical Observer* tells the following story: While attending a confinement he saw the nurse return, soon after the placenta had been expelled and taken from the room, with a small piece of meat, well peppered and salted, which the young mother ate. It was explained that "the meat was a piece of the afterbirth, taken to prevent after-pains; for that is how all animals do." The revolting morsel was given the credit for an early "getting up."

Well, why not? That would not be half so marvelous, to our thinking, as that the morsel stayed down.—*N. Y. Medical Journal.*

*This* takes the cake. [Editor.]

