# Correction

**Published:** 1898-07

**Authors:** 


Correction.—In the June number of The Homœopathic Physician at page 240, line 11 from the bottom, "concensus" should be spelled *consensus*. The same error occurs on page 245, line 20, and should be similarly corrected. Page 245, line 10, first word "with" should read *by*.

